# Temporal changes in cognitive functions and associated factors among stimulant users: a 12-month, prospective study

**DOI:** 10.1038/s41598-025-20028-3

**Published:** 2025-10-10

**Authors:** Albert Kar Kin Chung, Cheuk Yin Tse, Y. Doug Dong, Sau Wan Tang, Johnson Kai Chun Law, Edward Tin Kei Leung, Tommy Tsang Cheung

**Affiliations:** 1 Present Address: Department of Psychiatry, Li Ka Shing Faculty of Medicine, The University of Hong Kong, Hong Kong, Hong Kong; 2 Department of Medicine, Li Ka Shing Faculty of Medicine, The University of Hong Kong, Hong Kong, Hong Kong

**Keywords:** Stimulant use, Cognitive function, MoCA, FAB, Substance use disorder, Longitudinal study, Health care, Medical research, Risk factors

## Abstract

Cognitive impairments are commonly observed in individuals who use stimulants, yet few studies have tracked these individuals longitudinally. This prospective, 12-month longitudinal study investigated changes in cognitive functioning among active stimulant users and explored the associated factors. Adults with recent stimulant use were recruited from substance misuse treatment clinics and the community. Their demographics, history of drug use, and stimulant use disorder severity were assessed with structured clinical interviews. Global cognitive function and frontal executive function were measured every three months using the Montreal Cognitive Assessment (MoCA) and the Frontal Assessment Battery (FAB) over one year. Linear mixed-effects models evaluated temporal trajectories and associated factors related to the Changes in cognitive functions. Among 76 analysed participants, their frequency of stimulant uses were active and stable over 12 months. The MoCA scores averaged below the clinical cut-off at baseline, although no further persistent decline was observed. In contrast, FAB scores presented no systematic temporal changes. Being female and being of older ages were found to be associated with lower MoCA and FAB. None of severity, education, recent stimulant use, and lifetime duration of use, were found to be associated with cognition. While stimulant users exhibited some modest cognitive declines at baseline, no further substantial cognitive deterioration was observed over the one-year study period. Cognitive outcomes were more strongly associated with demographic factors than SUD severity or stimulant use patterns. These findings highlight the need for more sensitive tools to detect subtle cognitive changes associated with stimulant uses.

## Introduction

Stimulants constitute a broad class of sympathomimetic agents that are associated with dire potentials for addiction and dependence. Global seizures of amphetamine-type stimulants and cocaine had significantly surged and surpassed other drugs in 2022^1^. Currently, both methamphetamine and cocaine are the two most commonly used illicit psychotropic substances in Hong Kong^2^.

Methamphetamine uses are associated with potential profound cognitive disturbances. A meta-analysis of 17 cross-sectional studies revealed that chronic methamphetamine users had significantly lower cognitive scores in comparison to healthy controls, particularly in areas such as learning, executive functions, memory, and processing speed^3^. However, some studies did not find reliable differences in neurocognitive performances between methamphetamine users and controls^4–8^. Reviews on cocaine use also indicated impairments across executive functions and cognitive domains on attention and impulsivity^9,10^, but both domains were found to improve after a three-month abstinence.

Converging evidence indicated that hypofrontality is associated with cognitive impairments in chronic methamphetamine users^11^. Changes in frontal areas consistently showed lower fractional anisotropy by diffusion tensor imaging during the Wisconsin Card Sorting Test on attention, working memory and visual processing tasks^12,13^. Additionally, Oh et al.^14^ used structural MRI to demonstrate increased curvature of the genu, decreased width of the posterior midbody and isthmus on corpus callosum among methamphetamine users. The index of fractional anisotropy on corpus callosum also displayed correlations with performances in the Stroop Interference Task on selective attention, cognitive flexibility and processing speed^15^. The impacts of cocaine use on inhibitory-control-related activations and attentional control are evidenced by the reduced engagement of the amygdala-striatal, middle-frontal and right-frontoparietal networks in cocaine users, while negative toxicology results and abstinence were associated with increased activity in these networks^16^.

Longitudinally, Salo and colleagues^15^ found that the duration of methamphetamine use was linked to poorer performance on the Stroop Task in methamphetamine-dependent adults. Vonmoos and colleagues^17^ reported sustained and increased cocaine use was associated with poorer working memory over the span of one year; but for those with decreased use, small but non-substantial cognitive improvements were observed. In another two-year prospective study^18^, improvements in global cognition were observed for abstained methamphetamine users, although the magnitude was small and impairments were still indicated compared to the established normative range. Nevertheless, other studies correlating the duration of methamphetamine use with cognitive performances in chronic methamphetamine users failed to show meaningful associations^19–23^. More recently, using established task-based paradigms, Fitzpatrick and colleagues^24^ failed to find any meaningful behavioural signatures between methamphetamine users and healthy controls over six weeks.

The mainstays of treatment for stimulant use disorders (SUD) rely on contingency management, motivational interviewing, and cognitive behavioral therapy^25,26^. Adequate cognitive and executive functions are thus crucial to improve treatment retention and abstinence-related outcomes^27^. To date, there has been limited research presenting evidence on the longitudinal change in cognitive functions among stimulant users^17^, and whether different severity of SUD, degree of stimulant exposure, and other demographic factors are associated with potentially declined cognitive functions. We therefore conducted a 12-month prospective study to investigate the temporal changes in cognitive functions and to identify potential factors associated with the cognitive changes.

## Methods

### Design and participants

This was a prospective, longitudinal study that aimed to investigate temporal Changes in cognitive functions among stimulant users for 12 months. Participants were recruited through convenience sampling from the community, counseling centers for substance misusers, and substance misuse treatment clinics. Predetermined inclusion criteria required participants to be between 18 and 65 years old, with a history of repeated stimulant use for more than six months in the past year, and to have actively used stimulants within 28 days prior to enrollment. The stringency on the duration and recency of stimulant use ensured that individuals with current and sustained stimulant use were included. Participants were deemed ineligible if they used non-stimulant substances as their primary drugs of use, were taking regular prescribed medications, or had been diagnosed with neurodevelopmental or other psychiatric disorders according to the International Statistical Classification of Diseases and Related Health Problems 10th Revision (ICD-10) by cross-referencing to their electronic medical record, as they could confound cognitive changes related specifically to stimulant use. Each participant underwent face-to-face structured interviews every three months for a total of five assessments. After each assessment, participants received US$30 as an honorarium. All participants provided written informed consent for their participation. The study was approved by the Institutional Review Board of the University of Hong Kong/Hospital Authority Hong Kong West Cluster (HKU/HA HKW IRB reference number: UW 19–616), and the study protocol adhered to the Declaration of Helsinki.

### Measures

Demographic information for each participant including age, gender, education, and history of substance use were collected. Participants reported their highest level of education attained, which was converted to numerical numbers. Duration of stimulant use was reported in the unit of months since first use. Severity of SUD, frequency of stimulant use, and cognitive functions were assessed at baseline and every three months. The assessment of SUD severity was conducted by a board-certified psychiatrist and the rest of assessments were administered by the board-certified psychiatrist and trained research staff who were bilingual in Chinese and English.

*DSM-5 SUD Severity*. Board-certified psychiatrist (author AC) assessed each participant’s severity of SUD using the Structured Clinical Interview for DSM-5 Disorders – Clinician Version at each follow-up. The assessment identified 11 possible symptoms. The total number of symptoms endorsed ranged 0–11, with a greater number of symptoms indicating more severe SUD. Severity classifications were defined as follows: no SUD (0–1 symptom), mild (2–3), moderate (4–5), and severe (6 or more). In the case of participants used both methamphetamine and cocaine, the primary substance with higher numbers of symptoms was taken as the indicative SUD severity. Over the study period, it is possible for the severity of stimulant users to improve, worsen, or maintain at the same level across repeated assessments.

*Frequency*. Participants self-reported their estimated frequency of drug use using the Beat Drugs Fund Question Set 6 “Drug use frequency in the past three months”^23^ for a list of 12 commonly used substances including methamphetamine and cocaine as well as cannabis, heroin, ecstasy, ketamine, ice, methaqualone, zopiclone, and codeine. The specific wording for probing the frequency of use for each substance was, “In the past 3 months, how many times have you used the following substances?”, with possible responses of “Never”, “Used occasionally (specify number of times)”, or “Used regularly (specify times/day or times/week)”. In the current analysis, frequency was converted to an approximately standardized unit of active days of use within the past 90 days.

*Urine drug test*. A urine drug test was administered at the beginning of each visit with rapid immunoassay urine drug test kits (Multi Drug Screen Dip Panel, ultimed Products, Germany) to detect the presence of 10 commonly used substances, including 3,4-methylenedioxy methamphetamine, amphetamines, barbiturates, benzodiazepines, cocaine, ketamine, methadone, methamphetamine, opiates, and tetrahydrocannabinol. A positive urine stimulant test suggests a recency of stimulant use. The result of the urine drug test was coded binarily to reflect the presence of any stimulants.

*Montreal Cognitive Assessment (MoCA)*. The global cognitive function of the participants was assessed by paper-format MoCA, in both Chinese^28^ and English (v7.1)^29^ depending on the native language spoken by participants. MoCA had been previously validated as a quick screening tool for identifying gross cognitive impairment in individuals with substance use disorders with high sensitivity and specificity^30^. A score of < 26 was employed as the clinical cut-off to identify potential impairments in global cognitive functioning^29^.*The Frontal Assessment Battery (FAB)*. FAB has been used previously to assess the general frontal executive function of individuals with substance dependence^31,32^. It consists of six items that are implicated with various neural networks: conceptualization appears to be associated with dorsolateral frontal areas, mental flexibility to the prefrontal dorsolateral cortex and medial frontotemporal cortex, motor programming with the right prefrontal dorsolateral cortex and basal ganglia, and inhibition, interference control and autonomy with orbitomedial areas^33–35^. The validated Chinese translation was used for the majority of non-native English speakers in the study^36^. A cut-off score of 12 being used to probe potential impairments in frontal cognition.

### Statistical analysis

Demographic information was summarized as means and standard deviations for continuous variables and counts with percentages for categorical variables. Linear mixed-effects models were deployed to evaluate the trajectory of Changes in frequency of use and cognitive functioning among stimulant users of varying severity over the 12-month study period. Fixed effects were specified for time, DSM-5 defined SUD severity, and their interaction. Additional fixed effects for gender, age, years of educations, urine drug test, and lifetime duration of stimulant use were included to examine demographic and behavioral factors associated with cognitive functioning. These factors were not specified for the frequency model due to high collinearity among frequency, urine test, and lifetime use. Random effects only included random intercepts for each participant, but not for random slopes due to little explained individual variance. Time was entered as a continuous variable in the unit of month (range 0–12) with baseline (Time = 0) serving as reference. DSM-5 defined SUD severity was contrast-coded (simple coding) with a diagnosis of no SUD as reference. Binary factors for urine drug test (Negative = −0.5, Positive = 0.5) and gender (Female = −0.5, Male = 0.5) were also contrast-coded whereas continuous factors for age and duration of stimulant use were mean-centered. Thus, the interpretation of the regression results was based upon the intercept being the grand mean for all participants at baseline. No missing data were imputed as linear mixed-effects model could provide robust estimates assuming that the instances of missed sessions occurred randomly. Bayesian linear-mixed models of the same specified structure were also fit to afford further probabilistic interpretations of model estimates in relation to clinical cut-offs from MoCA and FAB. Bayesian results were reported with the mean and “Credible Interval” that represents the 95% highest posterior density regions of the posterior distribution. All models successfully converged, and model diagnostics were visually inspected. All model fitting were conducted using the lme4 (v1.1-35.5), lmerTest (v3.1-3), and brms (v2.22.0) packages for the programming language R (v4.4.1). All tests were two-tailed with a significance level ⍺ = 0.05, with no adjustments for multiple comparisons applied.

## Results

A total of 95 stimulant users consented and participated in this study. Most (88, 94.62%) participants were ethnic Chinese with only three participants completed interviews and assessments in English. However, 19 participants completed the baseline assessments only and were lost to follow-up; thus, 76 (80.00%) were included in the final analysis. The completion rates at each quarterly follow-up were relatively high, with 62 participants (81.57%) completed at Month 3, 56 (73.68%) at Month 6, 47 (61.84%) at Month 9, and 56 (73.68%) at Month 12.

### Demographics

**Fig. 1:**
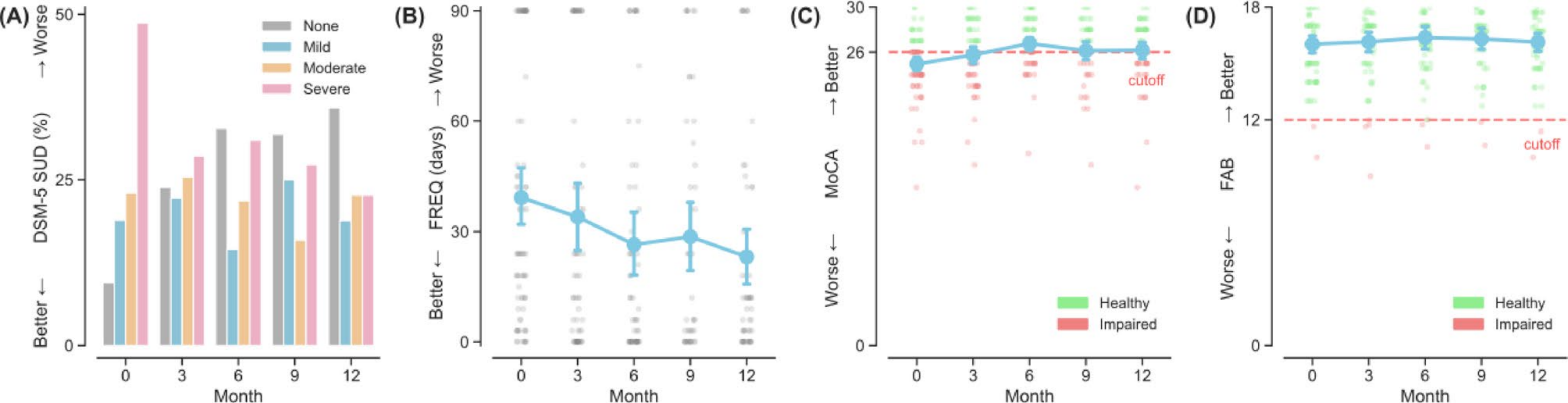
Temporal trajectories of DSM-5 severity, frequency of drug use, and cognitive functioning over the 1-year study period. Errorbars denote 95%CI. *Note*. CI: Confidence Interval; DSM-5: Diagnostic and Statistical Manual of Mental Disorders, 5th edition; FAB: Frontal Assessment Battery; FREQ: Frequency of use in the past three months; SUD: Stimulant User Disorder; MoCA: Montreal Cognitive Assessment.

Table 1 presents the participant demographics. The final analyzed sample comprised 76 participants with a mean age of 37.97 years (SD = 10.78), the majority of whom were male (68.4%). Participants had an average of 9.21 (SD = 2.76) years of formal education and overall half were single (52.6%). All participants had a history of methamphetamine and/or cocaine use with 78.9% and 46.1% using them within the three months prior to study enrollment, respectively. Most reported lifetime use of methamphetamine (84.2%) and cocaine (75%). Notably, 59.2% of participants reported using both substances, while only 6 participants (7.9%) identified as single drug user for methamphetamine or cocaine. The age of first methamphetamine use (mean = 21.5 years old, SD = 8.89) was relatively similar to that of cocaine (mean = 23.64 years old, SD = 7.98), and the average duration of lifetime methamphetamine use (mean = 148.69 months, SD = 108.62) was approximately twice as long as for cocaine (mean = 73.96 months, SD = 89.09). At baseline, the majority of participants were diagnosed with moderate (23.7%) to severe (47.4%) SUD, while 18.4% had mild SUD. 10.5% of the participants did not fulfil the DSM-5 SUD diagnostic criteria for having met only one criterion of stimulant use (Fig. 1A). The lifetime history of using other substance was common (see Table 1).

**Table 1:** Demographics of the final analyzed sample at baseline (*n* = 76).

Variables	Number (%)/Mean (SD)
Male	52 (68.42)
Age	37.97 (10.78)
Education (in years)	9.21 (2.76)
Marital status	
Single	40 (52.63%)
Married	14 (18.42%)
Divorced	21 (27.63%)
Widowed	1 (1.31%)
Active smokers	65 (85.53%)
Active drinkers	54 (71.11%)
Forensic records	47 (61.84%)
**Stimulant use status**	
Methamphetamine users	
Lifetime ever-use	64 (84.21%)
Current use within three months	60 (78.95%)
Age of first use	21.5 (8.89)
Duration of lifetime use (in months)	148.69 (108.62)
Cocaine users	
Lifetime ever-use	57 (75.00%)
Current use within three months	35 (46.05%)
Age of first use	23.64 (7.98)
Duration of lifetime use (in months)	73.96 (89.09)
Single drug users (methamphetamine or cocaine)	6 (7.89%)
Severity of SUD	
None	8 (10.5%)
Mild (2–3 symptoms)	14 (18.4%)
Moderate (4–5 symptoms)	18 (23.7%)
Severe (6 + symptoms)	36 (47.4%)
**Lifetime use of other substances**	
Cannabis	61 (80.26%)
Ketamine	57 (75.00%)
MDMA	52 (68.42%)
Zopiclone	28 (36.84%)
Heroin	27 (35.52%)
Note. FAB: Frontal Assessment Battery; MDMA: 3,4-methylenedioxymethamphetamine; MoCA: Montreal Cognitive Assessment; n: number of participants; SUD: Stimulant Use Disorder.	

### Frequency of stimulant use

**Fig. 2:**
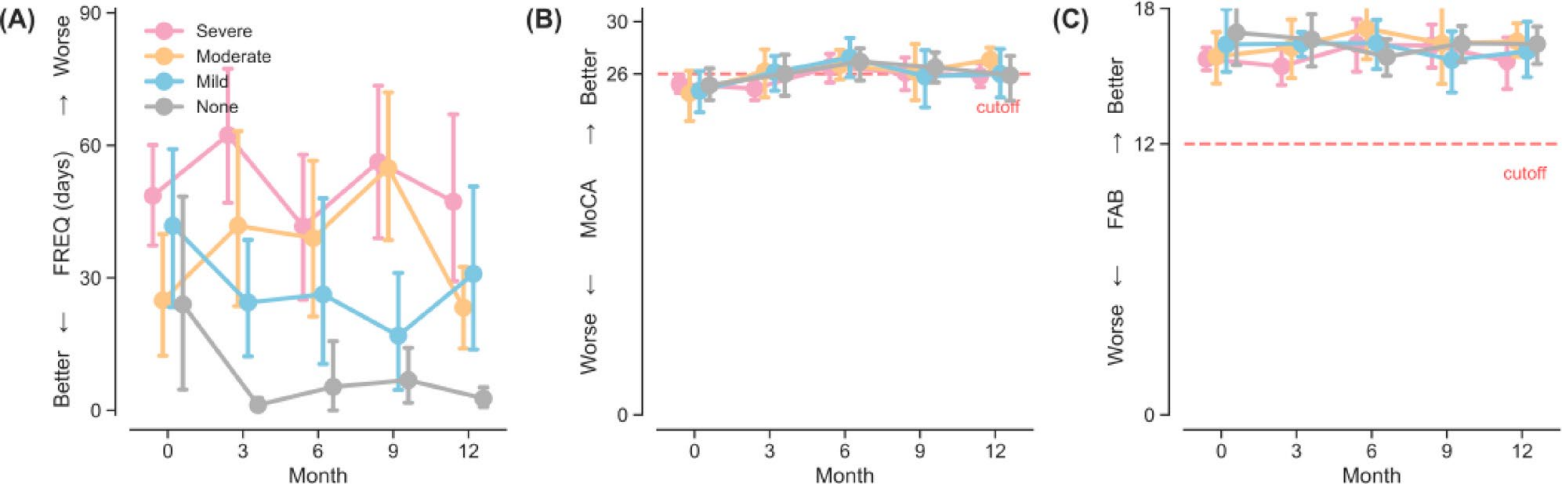
Temporal trajectories of outcome measures stratified by DSM-5 severity over the 1-year study period. Errorbars denote 95%CI. *Note*. CI: Confidence Interval; FAB: Frontal Assessment Battery; FREQ: Frequency of use in the past three months; MoCA: Montreal Cognitive Assessment.

At baseline, the reported days of stimulant use was estimated to be at least 33 days (95%CI = [26.86, 39.94], Table 2) in the past three months for an average participant controlling for all demographic and behavioral factors. Results from linear mixed-effects model, however, revealed no reliable changes in frequency of drug use over time as indicated by a non-significant fixed effect for time (B = −0.41, 95%CI = [−1.06, 0.24], *P* = 0.22, Fig. 1B). However, the reported baseline frequency of stimulant use in the past three months was associated with the severity of SUD. Specifically, those diagnosed with mild (B = 23.04, 95%CI = [6.73, 39.34]), moderate (B = 17.51, 95%CI = [2.03, 33.00]), and severe (B = 35.15, 95%CI = [21.04, 49.26]) levels of SUD all had at least 17 more days of stimulant use (all Ps < 0.05) on top of the grand baseline mean. Non-significant interactions between time and DSM-5 severity (all Ps > 0.29) suggest that stimulant users of varying symptom severity did not systematically differ in their frequency of use over time, meaning that, for all participants regardless of severity, their frequency of drug use did not increase nor decrease but maintained relatively stable over the 1-year study window (see Fig. 2A). That is, the participants actively and frequently used stimulants over the study period.

**Table 2:** Results of linear mixed-effects model predicting frequency of use and cognitive functioning over the 1-year study period.

	Frequency			MoCA			FAB		
	B	95%CI	P	B	95%CI	P	B	95%CI	P
Intercept (baseline mean)	33.40	26.86–39.94	**< 0.001**	24.78	24.15–25.42	**< 0.001**	15.93	15.48–16.39	**< 0.001**
Time (in month)	−0.41	−1.06–0.24	0.216	0.08	0.03–0.13	**0.001**	0.00	−0.04–0.04	0.976
DSM-5 (Mild – None)	23.04	6.73–39.34	**0.006**	0.21	−1.04–1.47	0.737	0.64	−0.46–1.75	0.253
DSM-5 (Moderate – None)	17.51	2.03–33.00	**0.027**	0.59	−0.61–1.79	0.335	0.13	−0.94–1.19	0.814
DSM-5 (Severe – None)	35.15	21.04–49.26	**< 0.001**	0.11	−0.99–1.21	0.846	−0.36	−1.34–0.61	0.465
Time × DSM-5 (Mild)	−0.34	−2.38–1.71	0.747	−0.07	−0.22–0.09	0.387	−0.09	−0.23–0.05	0.221
Time × DSM-5 (Moderate)	1.06	−0.91–3.03	0.290	−0.01	−0.16–0.14	0.891	−0.01	−0.14–0.13	0.906
Time × DSM-5 (Severe)	−0.35	−2.17–1.47	0.704	0.05	−0.09–0.19	0.464	0.05	−0.08–0.17	0.475
Gender (Male – Female)	–	–	–	2.31	1.12–3.50	**< 0.001**	1.11	0.32–1.91	**0.006**
Age	–	–	–	−0.07	−0.13 – −0.02	**0.014**	−0.04	−0.08 – −0.00	**0.039**
Education	–	–	–	0.19	−0.01–0.39	0.058	0.10	−0.03–0.23	0.139
Urine (Pos – Neg)	–	–	–	−0.22	−0.82–0.39	0.481	−0.13	−0.66–0.39	0.619
Duration of lifetime use	–	–	–	0.00	−0.01–0.01	0.896	−0.00	−0.00–0.00	0.962

Note. CI: Confidence Interval; DSM-5: Diagnostic and Statistical Manual of Mental Disorders, 5th edition; FAB: Frontal Assessment Battery; MoCA: Montreal Cognitive Assessment.Bold values correspond to statistical significance with alpha <0.05.

### Changes in the global cognitive function by MoCA

**Fig. 3:**
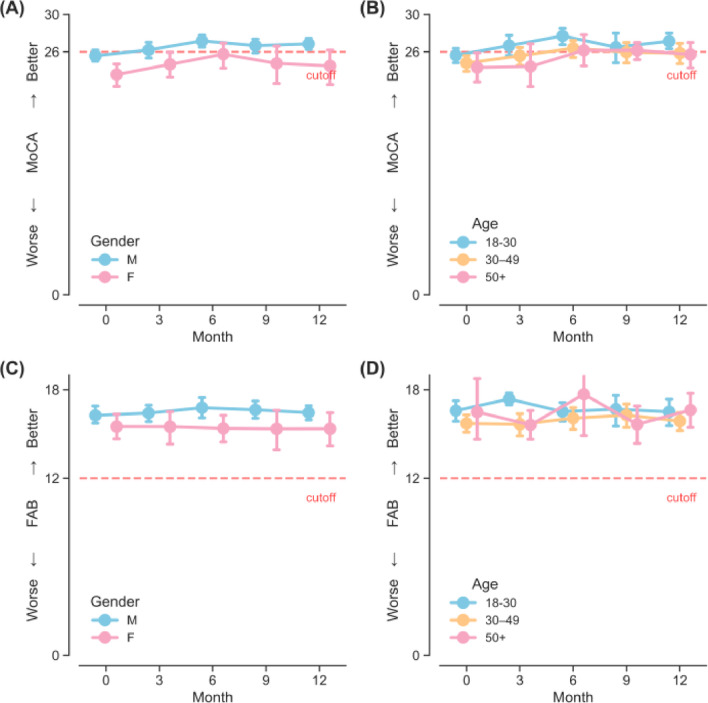
Differences in cognitive functioning stratified by gender and age. Errorbars denote 95%CI. *Note*. CI: Confidence Interval; FAB: Frontal Assessment Battery; FREQ: Frequency of use in the past three months; MoCA: Montreal Cognitive Assessment.

A participant of average behavioral and demographic characteristic was estimated to score 24.78 (95%CI = [24.15, 25.42], Table 2) on MoCA at baseline. Using the cut-off of 26, Bayesian analyses revealed that the probability of the grand mean of MoCA at baseline falling below 26 was 100%, illustrating potential impairments in global cognitive function among stimulant users as an entire group. The results from linear mixed-effects modelling revealed a small, positive fixed effect for time (B = 0.08, 95%CI = [0.03, 0.13], *P* = 0.001, Fig. 1C), of which the minute improvement was likely due to the practice effect of repeated measurements without parallel forms. Bayesian analyses provided additional information that at Month 12 the grand mean of MoCA was estimated at 25.71 (95% Credible Interval = [25.03, 26.35]), with its 79.62% of chance falling short of the cut-off of 26; that is, evidence of persistent cognitive impairment for stimulant users was clear despite the linear increases of small magnitude in MoCA over time. The fixed effects for DSM-5 SUD severity and MoCA revealed no reliable associations (all Ps > 0.39, Fig. 2B), and similarly, no evidence for interaction effects between time and DSM-5 SUD severity were found (Time × DSM-5: all Ps > 0.42, Fig. 2B). These null results indicated that participants with more severe SUD did not present greater impairments (nor improvements) in global cognitive function, nor did they show a systematically different trajectory than others over time. Nevertheless, female stimulant users averaged 2.31 points lower (95%CI = [−3.50, −1.12], *P* < 0.001, Fig. 3A) than their male counterparts. Older ages were also significantly associated with global cognitive decline (B = −0.07, 95%CI = [−0.13, −0.02], *P* = 0.014, Fig. 3B). However, neither more years of education (*P* = 0.058), a positive urine stimulant test result (*P* = 0.48), nor the duration of lifetime stimulant use (*P* = 0.90) was reliably associated with MoCA.

### Changes in the frontal executive function by FAB

At baseline, FAB was estimated to average 15.93 (95%CI = [15.48, 16.39], Table 2) for all stimulant users as a group, and Bayesian analyses estimated that its probability of falling below the cut-off of 12 was zero. Linear mixed-effects modelling discovered a non-significant fixed effect for time (*P* = 0.98, Fig. 1D), DSM-5 SUD severity (all Ps > 0.25, Fig. 2C), nor their interaction (all Ps > 0.22, Fig. 2C), suggesting the absence of reliable changes in FAB across SUD symptom severity or across time. Only being female (B = −1.11, 95%CI = [−1.91, −0.32], *P* = 0.006, Fig. 3C) and older ages (B = −0.04, 95%CI = [−0.08, −0.00], *P* = 0.039, Fig. 3D) were found to be significantly associated with a decline in FAB but not for education (*P* = 0.14), positive urine stimulant tests (*P* = 0.62), nor duration of stimulant use (*P* = 0.96). By Month 12, Bayesian analyses estimated that the grand mean of FAB averaged at 15.93 (95% Credible Interval = [15.41, 16.38]), with 0% of probability for falling under the cut-off of 12.

## Discussion

This prospective, longitudinal study investigated cognitive Changes in individuals with chronic and active stimulant use over a 12-month period. Analyses from linear mixed-effects modelling indicated that global cognitive function, as measured by MoCA, remained relatively static and below the clinical cut-off despite a slight increase over time. Frontal executive function, as assessed by the FAB, also showed no systematic temporal Changes across the study period. These findings suggest an absence of progressive cognitive deterioration among stimulant users over a 12-month period. There was also a lack of a dose-response relationship of stimulant use patterns—namely, DSM-5 SUD severity, or duration of use—on global and frontal cognitive performances.

The absence of associations between DSM-5-defined severity of SUD and cognitive outcomes—both at baseline and longitudinally—supports the view that routine clinical classifications may not effectively capture the neurocognitive burden of stimulant use^37^. Likewise, neither lifetime duration of stimulant use nor urine-confirmed recent stimulant use showed significant relationships with cognitive function. These null findings may reflect either a genuine dissociation between stimulant use patterns and gross cognitive performance in this population, or equally likely, the limitations in the sensitivity of global screeners like the MoCA and FAB to detect domain-specific or subtle deficits associated with stimulant use^10,38^.

In contrast, demographic factors including age and gender consistently predicted cognitive functioning. Older participants scored lower on both the MoCA and FAB assessments, reflecting well-established patterns of age-related cognitive decline that may be further exacerbated by neurotoxic effects of stimulant use^39,40^. Additionally, female stimulant users demonstrated lower MoCA and FAB scores, consistent with broader research suggesting gender-based differences in susceptibility to stimulant-related cognitive impairments^41,42^. These differences may stem from neurobiological or psychosocial factors. Future research should employ more rigorously matched demographic designs to determine whether stimulant use acts as a compounding risk factor—particularly among older adults and female users—accelerating cognitive decline when initiating stimulant use.

This study has several strengths. It is among the first long-term prospective investigations to track cognitive functioning in a sample of stimulant users who continued to use throughout the 12-month study period. The repeated assessments every three months allowed for fine-grained temporal analysis and helped clarify that cognitive functioning may not present persistent progressive decline. Additionally, the integration of urine drug testing and structured clinical interviews provided robust biological and behavioural corroboration to contextualize self-reported use.

However, several limitations must be acknowledged. The absence of a non-using control group restricts the ability to make definitive conclusions about the extent of cognitive impairment attributable to stimulant use. Practice effects from repeated assessments may have inflated follow-up scores, particularly for clinical screening tools like the MoCA and FAB that rely on paper-based static item formats^43,44^. Furthermore, comparing scores to normative data may be problematic, as many participants were likely to already exhibit cognitive deficits or long-term impairments prior to the study^45^. Another key limitation lies in the potential selection bias between treatment-seeking and non-treatment-seeking individuals^46–48^, within which treatment-seeking individuals may represent a subgroup with greater cognitive and motivational functioning, while non-treatment-seeking stimulant users may show more persistent cognitive decline over time that could not be realistically captured inherent to their attrition. Finally, recent use of task-based behavioral paradigms may have higher sensitivity and precision than paper-based assessments in detecting subtle changes in cognitive functions as a result of treatment outcomes^24,49^, which warrants future research.

## Conclusion

This prospective, longitudinal study reveals that global and frontal cognitive functions among stimulant users appeared relatively static over a one-year observed period. While SUD severity and stimulant use patterns were not reliably associated with cognitive changes, demographic factors such as being female and being older showed consistent associations with greater cognitive declines. These findings suggest that current diagnostic classifications may not fully account for the cognitive variability observed in stimulant users, or that standard paper-based clinical screening tools for cognitive changes may lack the sensitivity to detect the more nuanced, addiction-related cognitive disruptions. Thus, future research on developing and validating more sensitive paradigms to detect subtle cognitive changes in stimulant users are imperative.

## Data Availability

Individual participant data that underlie the results reported in this article after de-identification and the study protocol will be made available starting the date of publication and ending 36 months following the article publication to investigators whose proposed use of the data has been approved by an independent review committee for individual participant data meta-analysis. Proposals should be directed to the corresponding author AC and data access would follow the requirements laid in the Policy on the Management of Research Data and Records by the University of Hong Kong.
